# Balancing the blue economy and multiple stressor management in marine spatial planning at the land-sea interface

**DOI:** 10.1038/s44183-026-00192-3

**Published:** 2026-03-19

**Authors:** Ramesh Wilson, Sarah Reiter, Catarina Frazão Santos, Tundi Agardy, Lisa M. Wedding

**Affiliations:** 1 Department of Biology, University of Oxford, Oxford, UK; 2 Anderson Cabot Center for Ocean Life, New England Aquarium, Boston, MA USA; 3 Department of Animal Biology, Faculdade de Ciências, Universidade de Lisboa, Lisbon, Portugal; 4 MARE–Marine and Environmental Sciences Centre, ARNET–Aquatic Research Network, Universidade de Lisboa, Lisbon, Portugal; 5 School of Geography and the Environment, University of Oxford, Oxford, UK; 6 Sound Seas, Bethesda, MD USA

**Keywords:** Ecology, Climate sciences, Ecology, Environmental sciences, Ocean sciences, Geography, Environmental studies, Geography

## Abstract

Coastal ecosystems face complex, interacting stressors that challenge conventional management strategies. We propose a transformative, eight-component framework considering Marine Spatial Planning, Blue Economy objectives, and multiple stressor management at the land-sea interface. This framework employs adaptive, data-driven management, holistic ecosystem-based approaches, and stakeholder collaboration to mitigate cumulative impacts across multiple scales. Using Massachusetts (USA) as a case study, we hypothesise ways to apply this framework, enhancing coastal resilience and sustainability.

## Introduction

Coastal ecosystems are among Earth’s most productive and dynamic environments, boasting unique ecological processes and high biodiversity at the land-sea interface. They are crucial for nutrient transfer, coastal erosion control and infrastructure protection, pollution mitigation, and support both terrestrial and marine food webs^1,2^. Vegetated coastal habitats such as mangroves, salt marshes, and seagrass beds stabilise shores by attenuating wave energy, maintaining hydrological balance, trapping sediments, and filtering contaminants from runoff and industrial discharge^3,4^. At the same time, the biodiversity of coastal ecosystems is significant, and is supported by heterogeneous environments that foster diverse ecological communities adapted to fluctuating conditions such as periodic tidal submersion and emersion driving thermal stress, desiccation risk and wave exposure^4^. Economically, the ocean underpins fisheries, tourism, transport, and energy sectors, contributing roughly $2.6 trillion USD annually to the global blue economy^5^. As defined by the World Bank, the Blue Economy refers to the ‘sustainable use of ocean resources for economic growth, improved livelihoods, and jobs while preserving the health of ocean ecosystems’. With projections indicating that 50% of the world’s population will live within 100 km of the coast by 2030, human impacts and resource uses are expected to intensify^6^.

Coastal ecosystems, and their delivery of valuable goods and services, are inherently variable, experiencing natural fluctuations driven by tidal regimes and seasonal changes. This natural variability can obscure cause-effect signals; distinguishing anthropogenic impacts from background fluctuations therefore requires appropriate baselines and study designs (e.g. ‘Before-After Control-Impact’ designs, environmental gradients), and where possible, experiments that explicitly incorporate environmental variability^7–9^. Furthermore, coastal ecosystems become especially susceptible to degradation due to the cumulative impacts of multiple stressors occurring simultaneously or sequentially, both from local sources such as urbanisation, industrialisation, and agriculture (leading to habitat loss, pollution, and eutrophication)^10^, and global-scale pressures including climate change, sea-level rise, ocean warming, acidification, and changes in ocean circulation, resulting in shifts in species distributions and losses in ecosystem functions and ecosystem service provision^11^. Multiple stressors interact in complex and often unpredictable ways, further impacting ecosystems^12,13^. In broad terms, the effects can be considered additive (sum of individual impacts), synergistic (greater than sum of impacts), or antagonistic (less than sum of impacts), significantly altering ecosystems in unpredictable ways in the case of non-additive antagonisms and synergisms^12–15^. This poses challenges for management interventions, requiring a comprehensive understanding of how stressors at both the local and global scales interact against a backdrop of variability at the land-sea interface, to ensure interventions are effective across different spatial and temporal scales^15^.

Despite their ecological importance and vulnerability, coastal ecosystems remain underrepresented in multiple stressor policy and management frameworks. The 2022 UNESCO Summary for Policymakers on Ocean Multiple Stressors primarily addresses open-ocean and seafloor habitats but overlooks coastal features such as strong salinity gradients, sediment dynamics, and the unique marine-terrestrial connectivity that shapes biodiversity and ecosystem function^16^. Major international conventions such as the Convention on Biological Diversity (CBD)^17,18^, the United Nations Convention on the Law of the Sea (UNCLOS)^19^, the United Nations Framework Convention on Climate Change (UNFCCC)^20^, and the Paris Agreement^21^, offer broad environmental mandates but rarely address coastal zones explicitly or account for non-linear stressor interactions (see Supplementary Table 1). This gap partly reflects jurisdictional caution, since coastal waters fall under national sovereignty; however, it can neglect ecological connectivity and transboundary impacts, undermining ecosystem-based management across administrative boundaries^22,23^. Furthermore, marine conservationists have historically emphasised the distinctiveness of marine ecosystems, leading to institutional fragmentation between marine management and terrestrial watershed management^24,25^. For example, in the European Union, separate frameworks like the Marine Strategy Framework Directive^26^ (MSFD; achieving ‘Good Environmental Status’ in marine waters) and the Water Framework Directive^27^ (WFD; achieving good ecological and chemical status in inland, transitional and coastal waters through river-basin management plans) coexist, with differing scopes, indicators and reporting cycles that can complicate coordination at the land-sea interface^28^. Explicitly bridging terrestrial and marine governance within spatial planning frameworks is essential to effectively manage cumulative stressors across the land-sea interface^29^.

**Fig. 1:**
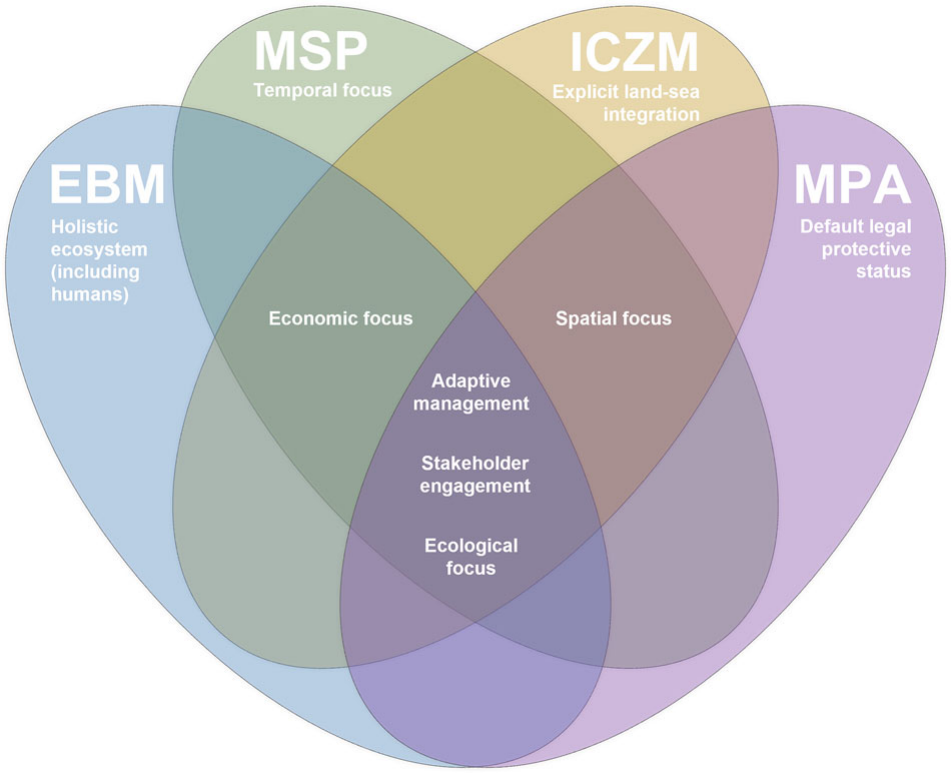
Four-set venn diagram illustrating overlaps in core aims of key coastal management approaches. EBM ecosystem-based management, MSP marine spatial planning, ICZM integrated coastal zone management, MPA marine protected area.

Marine spatial planning (MSP) is a critical tool for organising and managing marine spaces and has the potential to foster integrated management while balancing competing activities, reducing sectoral conflicts, and protecting ecosystem services^30,31^. MSP’s focus on the spatial and temporal distribution of activities differentiates it from other coastal management approaches, making it suitable for addressing multiple stressors whose cumulative impacts and interactions may vary over time and space (Fig. 1). Although MSP requirements vary globally, within the European Union for example, the MSP Directive (2014/89/EU) explicitly requires that maritime spatial plans take account of land-sea interactions (Art. 6(2)(a); see also Arts. 4(2) and 7)^32^. More broadly, applying an ecosystem-based approach within MSP entails systematically considering such land-sea interactions, and where appropriate, aligning with complementary coastal frameworks such as Integrated Coastal Zone Management (ICZM; also known as Integrated Coastal Management (ICM)), ensuring connectivity of open ocean and coastal resources, uses, and stressors across space and time.

Implementing flexible, adaptive strategies that evolve with new scientific insights and dynamic coastal and estuarine conditions is crucial to safeguarding coastal ecosystems and values^8^. Additionally, adopting spatial planning approaches that promote sustainable economic benefits and integrate considerations across species, spatial, and sectoral domains into a comprehensive land-sea framework can enhance these strategies’ effectiveness^33,34^. Balancing the development of a blue economy with environmental and social sustainability is vital to ensuring the resilience of coastal ecosystems amid a backdrop of interacting stressors at the land-sea interface.

**Fig. 2:**
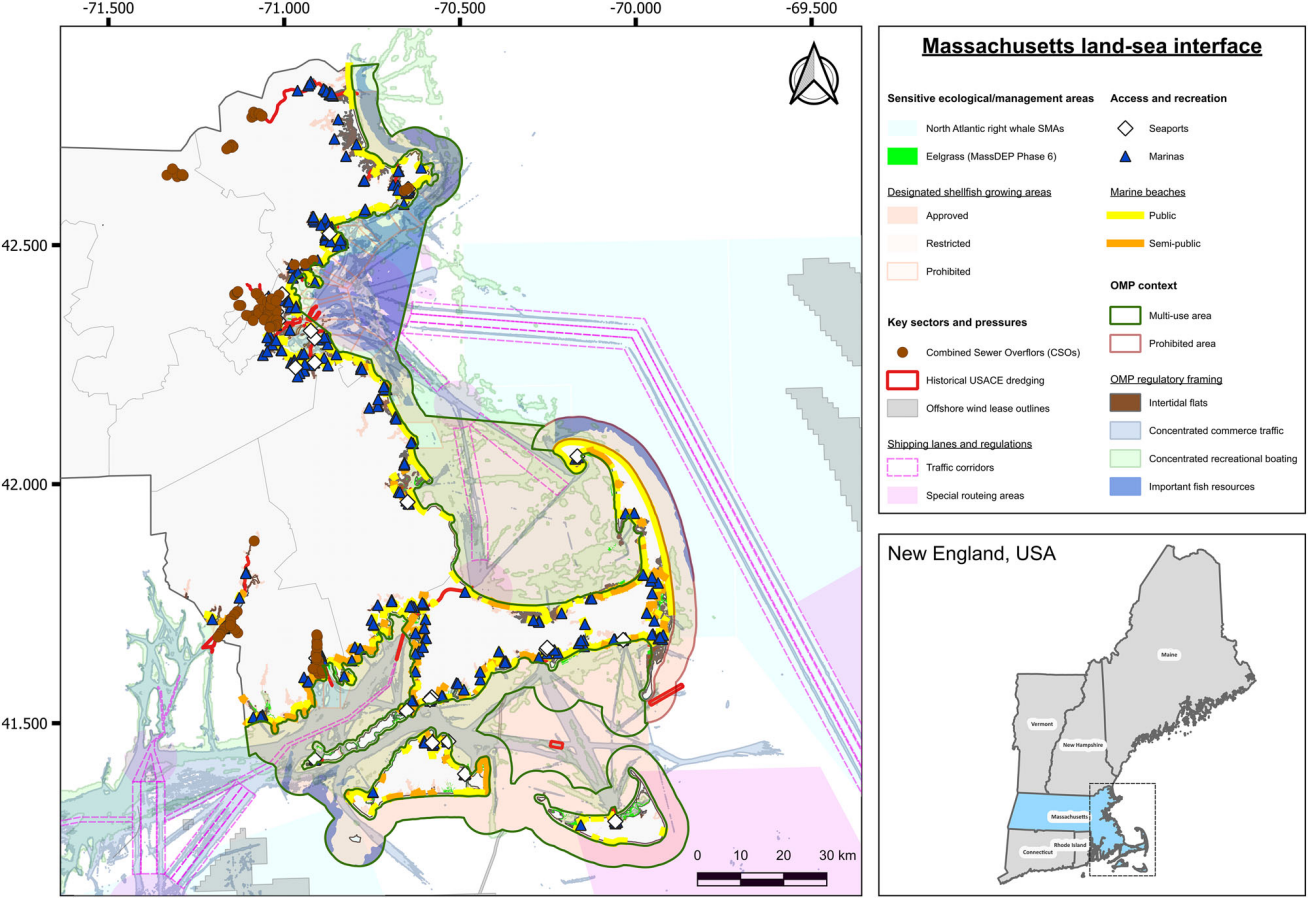
Activities at the Massachusetts land-sea interface. Illustrative map showing: (i) sensitive ecological/management features (North Atlantic Right Whale Seasonal Management Areas; eelgrass Phase 6; Designated Shellfish Growing Areas by status); (ii) key sectors/pressures (shipping corridors and special routeing areas per NOAA ENC; historical USACE dredge footprints; offshore wind lease areas; coastal CSO outfalls within 1 km of the shoreline); (iii) access and recreation areas (seaports; marinas; public and semi-public beaches), and; (iv) 2021 Ocean Management Plan (OMP) context (Prohibited and Multi-Use areas, Mask). Graticule labels show geographic coordinates (WGS 84; EPSG:4326) in decimal degrees, with longitude and latitude shown on the x- and y-axes, respectively. Right-hand inset locates Massachusetts within the wider New England region. Map created in QGIS (v3.40 LTR) from public-access geospatial feature services provided by the Commonwealth of Massachusetts (MassGIS/CZM, DMF, MassDEP, MassDOT; including OMP 2021 layers) and U.S. federal agencies (NOAA, BOEM, USACE). Data sources, service URLs, and licensing/permissions information are provided in the Supplementary Information.

Here, we propose a framework for balancing blue economy principles alongside multiple stressor management in MSP at the land-sea interface. Taking inspiration from the key components of climate-smart MSP defined by Frazão Santos et al.^35^, our framework components specifically address stressor interactions at the coastal interface, aligning sustainable economic activities with ecological resilience. Each component follows specific entry points aligned with broader UNESCO MSP guidelines^36^, to facilitate their operationalisation (Supplementary Table 2)—both at the beginning of, and during the MSP planning and implementation process. We demonstrate this approach through a Massachusetts (USA) case study, identifying practical pathways, challenges, and opportunities for integration within an existing sub-national policy context. Because the United States has no federal statute mandating MSP, and national direction has come via executive policy that has shifted over time (e.g. Executive Order 13547 (2010)^37^ promoted coastal and MSP, later superseded by Executive Order 13840^38^ without an MSP mandate), MSP practise is largely state-led and supported by federal data infrastructure (e.g. the NOAA-BOEM platform). We therefore draw recommendations from existing international examples, including the EU MSP framework, as clear reference points, while synthesising within the context of Massachusetts as an illustrative sub-national case study (see Supplementary Box 1 for further case-study rationale and blue economic context, and Fig. 2 for illustrative map of key sectors, coastal activities, and potential overlaps at the land-sea interface). We further provide a Research Brief for Massachusetts that distils the case-study into a short, practitioner-oriented summary for policymakers and planners (see Supplementary Materials).

These components are designed to: (1) promote a holistic approach to managing multiple stressors; (2) ensure integration at the land-sea interface within management frameworks, avoiding gaps that could arise from separating terrestrial and marine considerations; (3) facilitate cross-sectoral collaboration to support long-term sustainability within blue economy objectives.

## Balancing the blue economy and coastal multiple stressor management

MSP typically incorporates economic considerations through sectoral coordination, often aligning implicitly or explicitly with goals emphasised by blue economy initiatives^39,40^. Initiatives like the High-Level Panel for a Sustainable Ocean Economy, comprising 18 nations representing 50% of global coastlines, aim to protect 30% of the ocean by 2030 and sustainably manage 100% of national ocean areas^41^. At the European level, the 2025 European Ocean Pact has committed €1 billion towards growing a competitive, sustainable blue economy by tightening links between marine planning, investment and open data—explicitly inviting multidisciplinary collaboration across ecology, economics, technology, and governance^42^. Recent legal and policy signals also push the blue economy toward ‘nature-positive’ and regenerative practice, including the UN High Seas (BBNJ) Agreement reaching 60 ratifications in September 2025^42^, the ITLOS May 2024 advisory opinion clarifying States’ duties under UNCLOS to prevent, reduce, and control marine pollution^43^, and the ICJ’s advisory opinion in July 2025 clarifying climate-related obligations of states^44^. However, large-scale blue economy strategies frequently prioritise national economic interests (e.g. GDP growth through offshore wind or industrial port development), which may not always align directly with state-level or community-scale interests^45^. Additionally, many coastal and marine systems function as open access commons, and without mechanisms such as ocean-assets trusts to ensure shared stewardship, private investment risks exploitative, unsustainable use, and exclusion of traditional users^46^. Recognising and bridging this gap by harmonising national priorities with local economic sustainability and ecological resilience is essential for effective coastal management, in the face of multiple interacting stressors operating at multiple scales.

MSP and blue economy initiatives already share significant conceptual alignment in their goals of balancing economic activities with sustainable management of marine resources^47^. The blue economy’s sector-specific economic focus offers opportunities to promote enhanced monitoring and data collection^48^. Leveraging this could provide practical entry points to integrate sectoral management more explicitly into multi-sectoral MSP frameworks, allowing systematic evaluation of cumulative environmental impacts and enabling more informed management at the land-sea interface.

Despite conceptual alignment, practical approaches often remain fragmented across sectors^49,50^. When blue economy sectors are managed independently rather than integrated into a cohesive MSP framework, economic activities may inadvertently exacerbate ecological impacts^51,52^. Without a data-driven understanding of the non-linear impacts of multiple pressures, management actions at the local level may be ineffective or even harmful. The proposed framework of mutually reinforcing components for balancing blue economy goals alongside multiple stressor management addresses this challenge, optimising the efficacy of MSP initiatives at the land-sea interface. The components are detailed below, together with their potential application to the Massachusetts case study.

### Component 1: Promoting research to understand stressor interactions at the land-sea interface

Modern coastal management demands a sophisticated understanding of multiple, interacting stressors. These stressors often produce non-linear, multi-directional ecological effects as resource use intensifies, with frequent non-additive outcomes across studies^12,53^. To make responses more predictable and management-ready, we require standardised definitions and terminology, alongside comparable study designs and integrative modelling^13^. Moreover, because interaction signs and magnitudes are context-dependent across space, time and levels of organisation^12,13,54–57^, robust inference requires designs and baselines that resolve spatiotemporal structure and mechanism, complemented by experiments that explicitly incorporate environmental variability and extremes^7,56,58–60^.

Cumulative Impact Assessments (CIAs) can provide a foundation for this approach, enabling planners to evaluate how combined pressures linked to blue economy sectors—for example, nutrient/contaminant inputs from coastal development and aquaculture, underwater noise and vessel-strike risk from shipping and coastal ports, and benthic disturbance from dredging, cable installation and bottom-contact fishing - together with climate drivers, may exceed ecological thresholds across the land-sea interface^61–64^. However, in many implementations, such as Sweden’s Geographic Information System (GIS) tool ‘Symphony’ which is used nationally and shared regionally across the Baltic and North Sea, the CIA is functionally additive: mapped pressure layers and ecosystem components are paired via a sensitivity matrix, and cumulative impact in each grid cell is calculated as the sum of the products^51,65,66^. The assumption of additivity, however, is a core limitation as responses to multiple stressors are seldom linear. Accordingly, thresholds and sensitivities should be empirically grounded and periodically revisited, and CIAs should integrate spatial and temporal information on physical, ecological, and socioeconomic variables across the land-sea interface; where feasible, mapping should be paired with interaction-aware analyses so that non-additive combined effects are not missed^61,67,68^.

To be integrative and holistic, it is crucial to embed CIAs and Strategic Environmental Assessments (SEAs) within a broader, systems‑science framework. Systems thinking tools such as Sankey diagrams can support visualisation of stressor pathways and energy or material flows^69^, although often show limitations in depicting dynamic feedback^70^. Furthermore, AI-driven platforms like ARIES (ARtificial Intelligence for Ecosystem Services), demonstrate how semantic modelling and machine‑learning can dynamically integrate diverse datasets and forecast socio‑ecological outcomes in complex coastal systems^71^. By coupling traditional assessments with systems‑science approaches and AI tools, MSP can more effectively capture the full complexity of multiple stressor interactions, enabling truly adaptive, data‑driven decision‑making at the land‑sea interface.

#### How Massachusetts could operationalise Component 1

Leveraging this approach in Massachusetts could streamline efforts to map overlapping land-sea pressures in the adjacent Gulf of Maine, guiding protected zone designations and advancing the state’s blue economy goals. Although the state employs forward-looking coastal policies, significant gaps remain in addressing the interactive, non-additive effects of stressors within planning evidence and management frameworks. The Gulf of Maine is one of the fastest-warming bodies of water globally^72^. This warming, alongside associated climate-change stressors such as acidification, and direct coastal pressures such as contaminants and nutrient pollution, presents potential interactive stressor effects that are yet unquantified, but could profoundly impact ecosystems and dependent industries at the land-sea interface.

To make this tractable at the land-sea interface, Massachusetts could couple the state Ocean Management Plan (OMP: Volumes One^73^ and Two^73^) spatial layers available via MassMapper/MORIS^74^ with embayment nitrogen evidence from the Massachusetts Estuaries Project (MEP)^75^, and watershed planning outputs such as the Cape Cod Section 208 Plan^76^ to identify land-sea interaction research hotspots where land-derived nutrient loads intersect ocean warming/heatwave anomalies and intense use corridors (e.g. shipping and port activities). Such hotspots could then become candidates for mechanistic studies (e.g. eelgrass/shellfish performance under combined heat × eutrophication) and pilot cumulative impact analyses that extend beyond simple additive overlays. The Ocean Management Plan Science Framework^77^ already highlights commitments to iterative data acquisition and decision-support like comprehensive seafloor mapping and climate adaptation research; however it does not establish guidelines for cross-stressor analyses or predictive modelling of interactive effects across time and space. Massachusetts could further adopt a CIA-based GIS tool (e.g. Sweden’s *Symphony*) to compare planning scenarios, while explicitly pairing it with interaction-aware analyses derived from targeted research efforts, resolving aforementioned shortcomings in the tool’s current additive scope. More adequate predictive modelling based on this evidence across the land-sea interface would support the anticipatory ability of key blue economy sectors including tourism, fisheries, and aquaculture, to mitigate compounded stressor effects, facilitating effective zoning and adaptive operations in sensitive areas^78^.

### Component 2: Integrating across species, spaces, and sectoral domains

Effective coastal management must recognise the interconnectedness of ecosystems, where stressors across the land-sea interface impact species, habitats, and economic sectors in diverse ways. The ‘3S’ framework (Species, Spaces and Sectors) by Wedding et al.^34^ formalises this lens and offers a strategic approach to addressing multiple stressors on ocean systems. This framework emphasises that ecosystem health is influenced not only by individual stressors but by their combined effects across species (biodiversity, community/ecosystem dynamics), spaces (habitats, wider seascapes, migration corridors), and sectors (human activities, blue economy), revealing critical intervention points for policy and management.

Wedding et al.^34^ propose network modelling as a valuable tool, enabling managers to visualise and quantify relationships among stressors across the 3S domains. Network models provide a tractable way to identify priority areas by pinpointing ‘nodes’ most sensitive to stressor interactions, guiding effective conservation efforts or regulatory interventions. For example, in their Arctic case study, this approach anticipated compounded effects of global change stressors like climate change and local stressors such as increased shipping, enabling proactive management of vulnerable regions^34^.

#### How Massachusetts could operationalise Component 2

Massachusetts already has a strong foundation through the 2021 Ocean Management Plan (OMP) which delineates Prohibited and Multi-Use areas and operates on a five-year monitor-evaluate-adjust cycle^77^. There are opportunities to enhance multiple stressor management by adopting a holistic lens linking across the 3S framework. Building on this framework, the state could collaboratively apply a 3S lens explicitly at the land-sea interface to prioritise interventions where interacting stressors converge. Practically, this means assembling a ‘3S network’ from existing state and federal datasets (served via CZM’s MassMapper/MORIS tool^74^ and wider Mapping and Data Management Program^79^) so nodes (e.g. eelgrass, shellfish areas, SMAs) and edges (traffic density, historical dredging, coastal effluent) could be scored and tracked over space and time (see Fig. 2 for summary of key activities/habitats at the land-sea interface). Publishing these inputs as live services and simple dashboards lets planners and users visualise trade-offs and ecosystem services, and see where risk accumulates as conditions change.

Within this 3S network, species and habitats may include eelgrass beds (habitat-forming, stress-sensitive), Designated Shellfish Growing Areas (water-quality-dependent), and North Atlantic Right Whale Seasonal Management Areas (protected-species risk). Spaces may include OMP Prohibited/Multi-Use areas and water-dependent use (WDU) concentrations (e.g. commerce traffic, recreation). Sectors could further cover shipping corridors and special routing areas, offshore wind lease footprints, historic dredge footprints (legacy benthic alteration), and coastal CSO outfalls (land-derived inputs) (see Fig. 2). The network could be updated routinely as MassMapper/MORIS and agency partners refresh planning layers, keeping analyses aligned with current conditions. With this evidence base, managers can set clear, pre-agreed triggers that connect network ‘hotspots’ to familiar measures: seasonal/time-area tools (e.g. regional fishery closures) where risk peaks for particular species or habitats; speed management in SMAs when whale risk is elevated; and routing preferences, setbacks or buffer adjustments around mapped ‘Special, Sensitive, or Unique’ (SSU) resources (e.g. eelgrass) where cables, dredging or dense traffic intersect sensitive areas. Embedding these triggers in the OMP’s 5-year cycle keeps MSP zoning and operating conditions flexible. When the network shows strengthening linkages or new high-risk nodes, closures, speed measures or buffer changes could be proposed transparently, reviewed and, if warranted, implemented.

Collaboration could be formalised, for example, through a government-convened working group drawing in the Division of Marine Fisheries^80^ (DMF; fisheries/time-area rules), Department for Environmental Protection^81^ (MassDEP; water quality/CSOs), the Clean Energy Center (MassCEC)^82^, and port authorities (offshore wind planning/operations), alongside municipal partners to co-design mitigations and share monitoring data. Existing venues such as the Fisheries Working Group on Offshore Wind Energy^83^ provide a ready interface between fisheries and energy sectors and could review network outputs and advise on operational responses at the land-sea interface, making collaboration routine and tying decisions to shared, up-to-date evidence.

### Component 3: Explicitly incorporate bidirectional interactions at the land-sea interface

Effective coastal management requires taking explicit account of land-sea interactions (LSI) in both directions; how land activities affect marine systems and how sea processes and uses feed back on land^84^. This bidirectional framing is widely recognised in broader MSP guidance, such as the EU MSP Directive^32^ which lists LSI among the minimum requirements, and is transversal throughout the remit of our wider recommendations. Internationally, programmes such as the Netherlands’ Delta Programme and ‘Room for the River’ project combine principles of MSP and ICZM, providing models demonstrating LSI-aware planning, and link watershed management, flood risk reduction and coastal adaptation through combined spatial and engineering measures^85^; an approach that aligns with a truly bidirectional LSI lens.

Management approaches such as Integrated Coastal Zone Management (ICZM/ICM) traditionally address near-shore pressures (e.g. nutrient and pollutant runoff)^86^, yet connections to larger-scale ocean dynamics such as species migrations, currents, and sediment budgets can be missed if LSI is treated narrowly^84,87^. Evidence from freshwater systems shows that stronger habitat connections boost resilience to multiple stressors by supporting species movement and functional diversity^88^. Translating this resilience into practice across the land‑sea interface means designing spatial plans that jointly manage upland watershed uses, shoreline development, and marine zones to sustain continuous ecological flows and buffer against multiple stressors^51^.

#### How Massachusetts could operationalise Component 3

Massachusetts shares challenges echoed across many other states and countries, such as rapid sea‑level rise, urbanised watersheds, and competing coastal uses. Therefore, adopting a bidirectional LSI layer more explicitly into the OMP review cycle would help bridge current gaps between watershed actions and marine zoning. Practically, this could involve coupling land-to-sea accounting of nutrient and pollutant loads with sea-to-land measures for shoreline change, and integrating those indicators into the OMP’s 5-year monitor-evaluate-adjust process. The state has already made numerous efforts to account for terrestrial impacts on marine environments, including CZM’s Coastal Water Quality^89^ and Coastal Habitat programmes^90^, the ‘StormSmart’ Coasts toolkit^91^, and the MassMapper/MORIS portals^74^ that serve authoritative, map-ready layers to planners and municipalities. Using these platforms to publish a set of LSI indicators such as embayment nitrogen loads vs. ecological thresholds, shoreline-change and flood-risk metrics, and proximity of sensitive waters to outfalls, would make trends visible, incentivise focus on quantifying non-linear stressor interactions across the land-sea interface, and trigger timely management responses under the plan.

Regarding land-to-sea, OMP evidence could directly reference Cape Cod’s Section 208 watershed plan^76^ and MEP-derived nitrogen targets so that exceedances in embayments reliably flag adjacent marine uses (e.g. shellfish harvest areas, eelgrass SSUs) for review or mitigation; CZM grant programmes could then prioritise stormwater retrofits or nature-based treatments where marine sensitivity is highest. Additionally, for sea-to-land considerations, the ‘StormSmart’ suite already provides guidance and map tools for sea-level rise, flooding and erosion; codifying living-shoreline and beneficial reuse of dredged sediment options (e.g. thin-layer placement to sustain marsh elevation or beach nourishment with suitable material) as preferred measures in high-risk reaches would align shoreline permitting with downstream habitat resilience goals^91^. Embedding these bidirectional indicators in OMP’s governance loop keeps actions concrete and adaptive, operationalising existing frameworks across the land-sea interface and tying decisions to transparent, state-maintained data services and programmes.

### Component 4: Investing in data collection and data sharing

Effective coastal ecosystem management relies on accurate, timely, and interoperable data, especially at the land-sea interface where terrestrial pressures and marine responses must be analysed together. Robust, cross-domain data collection and sharing underpins MSP decisions about cumulative and interaction-aware stressors across ecological, social, and economic domains. In practice, this means co-registering both land-to-sea, and sea-to-land datasets at appropriate temporal frequencies so planners can detect non-linear, non-additive effects and act before thresholds are crossed. In addition to ecological reference points, such data collection could include socio-economic and cultural-use datasets (e.g. livelihood dependence, access/use intensity, cultural values, distributional/equity indicators) so managers can align user requirements to ecological sustainability, and maintain social licence. Without high-quality, standardised, multidisciplinary datasets, MSP risks blind spots that hinder resilience and sustainable growth within the blue economy^92^. Moreover, the issue of scale must be considered: many LSI indicators (embayment nitrogen, local shoreline change) are local, while MSP decisions are often at the spatial scale of state or regional sea, necessitating reconciliation of data scale and source. A pragmatic approach is a nested design; collect and QA data at local catchment/shoreline units, expose them via interoperable services with clear metadata, and aggregate upward to MSP planning units with reproducible methods. EU guidance on LSI and ecosystem-based MSP supports this workflow, further stressing multi-level governance, common standards, and iterative updates^93,94^.

Several operational models demonstrate elements of good practice. Portugal’s MSP Geoportal publishes maritime, environmental, and socioeconomic layers to support plan implementation and stakeholder access^95^. At the regional-sea, multilateral scale, the OSPAR Data and Information Management System (ODIMS)^93,94^ and its Assessment Portal provide standardised protocols, metadata, and web services to share North‑East Atlantic data across contracting parties, reducing duplication and improving transparency^96^. Likewise, the International Council for the Exploration of the Sea (ICES)^94^ maintains open data policies (CC BY 4.0) and thematic portals spanning biology, environment, oceanography, and underwater noise—data infrastructures that directly support cross-sector analyses and long-term trend assessment^97^. Taken together, these examples demonstrate the value of standards, licensing clarity, and live web services for MSP-relevant evidence.

#### How Massachusetts could operationalise Component 4

Massachusetts has a strong base in CZM’s Mapping and Data Management Program, with the MORIS/MassMapper portals providing authoritative, map-ready layers^74,79^. To make the system fully LSI-aware and interaction-ready, the OMP can foster cross-agency data sharing, and publish a small set of LSI indicators and multiple stressor data streams as live services with agreed standards. In practice, that means: setting protocols (licensing, metadata, QA/QC, update cadence) for integrating cross-agency streams such as MassDEP water-quality monitoring^75,81^, DMF fisheries/shellfish data^80^, and MWRA outfall data^98^, and receiving-water monitoring alongside coastal habitat/use layers in MORIS^74^; and ensuring indicators can be rolled up from local embayments and municipal shorelines to the OMP planning units used for zoning and review. MassDEP’s surface-water portals and MWRA’s long-running ambient/outfall monitoring programme already deliver time-series suitable for land-sea indicators (e.g. nutrient/contaminant loads, receiving-water responses)^81,98^. Publishing these via MORIS, cross-linked with DMF resources would allow planners to test spatio-temporal covariation between pollution events and ecological or catch trends, then supplemented with follow-up analyses (controlled field studies, mechanistic models, or multivariate time-series) to disentangle the relative influence of stressors over time and space^99^. Moreover, increasing the frequency and resolution of data collection across physical, biological, and socio‑economic parameters will improve predictive model robustness, while visualising interactive networks could generate testable hypotheses about stressor interactions, laying the groundwork for targeted experimental or modelling follow‑up to establish causality.

Across the sea-to-land direction, ‘StormSmart’-aligned shoreline datasets (erosion, flood risk) and coastal habitat status (eelgrass, marsh condition) are best published with consistent identifiers so they can be joined to municipal planning layers and OMP management areas, enabling routine scale reconciliation: local metrics inform state-level triggers in the OMP’s five-year monitor-evaluate-adjust cycle. To support transparency and reuse, mirroring ICES-style open licensing and OSPAR-style metadata and services could provide an operational stencil, alongside maintaining a single catalogue of LSI indicators in MORIS/MassMapper so municipalities, agencies, and sectors can all pull from the same source of information.

### Component 5: Prioritising adaptive, data-driven management

Adaptive monitoring and data-driven management are critical for handling the natural and anthropogenic variability of the land-sea interface and their associated stressors to ensure resilience and continuity of ecosystem services^100^. High-frequency, fine-scale data from remote sensing, electrochemical sensors, and IoT (Internet-of-Things) networks capture real-time fluctuations, establish ecological baselines and trigger management responses^101^. Additionally, while Long-Term Ecological Research (LTER) programmes provide indispensable context on enduring trends and regime shifts^102^, multi‑year analysis and publication timelines often limit their direct utility for immediate management decisions^102^. Instead, day‑to‑day adaptive responses typically draw on more immediate, management‑oriented monitoring, such as continuous water‑quality sensors, vessel‑tracking systems, and automated biological detectors, feeding live data into decision‑support dashboards^103,104^. In the Baltic Sea MSP, high-frequency monitoring programmes capture physical, chemical, and biological parameters, with data feeding into the Baltic Marine Environment Protection Commission’s (HELCOM) decision-making^105^. This allows adaptive adjustments, such as modifying shipping lanes to reduce pollution in sensitive areas. HELCOM’s stakeholder integration, including fishers and NGOs, enhances compliance and data accuracy through local insights^106^. This collaborative approach has improved the region’s capacity to manage multiple stressors effectively, demonstrating the importance of adaptive monitoring in sustaining coastal resilience^107^.

The temporal scale at which stressor interactions are assessed is crucial, as their significance and directionality can shift over time^12,59^. Additionally, the mode of stressor application, whether ‘press’ (continuous, chronic stress) or ‘pulse’ (episodic, acute stress), can also determine ecosystem responses, with press stressors often eroding resilience over time, while pulse stressors can cause abrupt but sometimes reversible shifts^108^. Moreover, pulse vs press disturbances have different management implications: pulse shocks may require more rapid, ephemeral responses; whereas press stressors erode resilience and call for more structural measures. Recognising these temporal dynamics ensures adaptive monitoring accounts for both short-term variability and long-term trends, improves detectability of shifts in interaction sign/magnitude, and more adequately informs spatial planning and decision-making.

#### How Massachusetts could operationalise Component 5

In Massachusetts, the CZM already coordinates map-ready evidence via MORIS/MassMapper^74,89^, but adaptive MSP at the land-sea interface needs those data streams to be integrated with clear objectives, measurable indices, and pre-agreed triggers so managers can adjust allocations in real time while staying aligned with environmental and economic goals. Practically, this means pairing pulse signals (e.g. DMF paralytic shellfish toxin thresholds that close harvest areas; MDPH beach bacteria advisories; dynamic NOAA right-whale speed zones) with press trends (e.g. MWRA outfall/receiving-water time series, buoy and HF-radar records) and publishing them as live layers in MORIS. When monitoring shows ecosystem function declining or risk peaking from overlapping activities, managers could then modify zoning or impose time-area restrictions (e.g. temporary operating conditions, dredging windows, aquaculture adjustments) and then relax them as indicators recover, with all actions reviewed through the OMP cycle. Investing in additional remote sensing and sensor networks strengthens this cadence, crucially supported further by governance integration: set update frequency, QA/metadata, and licensing for cross-agency feeds (CZM with MASSDEP/DMF/MWRA, and federal partners for protected-species/traffic), and define a compact dashboard of indices (e.g. biotoxin and faecal-indicator exceedances, marine heatwave intensity, protected species detections, and embayment water-quality status) so natural variability is distinguished from true stressor interactions. Furthermore, integrating diverse datasets into centralised systems enables timely information flow, supporting responsive, evidence-based decision-making^100^.

### Component 6: Enhancing stakeholder engagement and cross-sectoral collaboration

Engaging diverse stakeholders and fostering cross-sectoral collaboration across the land-sea interface is essential for effective MSP and blue economy integration amid multiple stressors. Because authorities and communities on land and at sea often work in different policy arenas and seldom meet in joint processes, effective land-sea integration requires intentional bridges between them. Incorporating perspectives from local communities, industry representatives, NGOs and government bodies enhances the legitimacy, acceptance, and inclusivity of management decisions across resource-dependent sectors^109,110^. Engagement mechanisms such as workshops, public consultations, and participatory mapping on shared data portals ensure stakeholders are actively involved in co-defining problems and options on both sides of the coastline^109^. Additionally, building capacity through education and training empowers stakeholders to engage effectively^111^. Crucially for multiple stressor management, deliberation should centre on cumulative and potentially non-linear interactions, so that stakeholders prioritise indicators, thresholds and triggers that make sense at local (embayment/shoreline) scales but roll up to MSP decisions. This involvement would further strengthen adaptive management and responses to multiple stressors by making stakeholders active partners in monitoring and adjusting strategies based on real-time insights and pre-determined LSI indicators.

A balanced blue economy needs social science alongside biophysical analysis. Social research surfaces distributional impacts, equity and justice concerns, cultural values, social licence to operate, and the governance/power dynamics that shape compliance and effectiveness. Using participatory mapping and co-production, plus tools such as social-network and conflict analysis, helps ensure MSP decisions are legitimate, implementable, and durable across the diverse needs of the land-sea interface^112,113^.

#### How Massachusetts could operationalise Component 6

In Massachusetts, CZM programmes like the Regional Program^114^ and Communications Program^115^ provide a foundation for interagency coordination and stakeholder engagement. There could be greater emphasis on convening shared-table land-sea processes that bring municipal/watershed authorities and offshore/marine users into the same room. Expanding these frameworks to involve a broader range of stakeholders from the private sector, local communities, and blue economy sectors presents several opportunities. For example, this could be fostered by joint LSI workshops co-chaired by municipalities and watershed groups alongside DMF, port authorities, and offshore-wind representatives (MassCEC/developers), using MORIS/MassMapper as the common, standard map for participatory discussion. The Regional Program, with locally embedded coordinators, supports community engagement on coastal issues^114^, and is well placed to co-host such cross-boundary sessions to surface conflicts and co-benefits of planned management activities across the land-sea interface. Expanding its reach with structured workshops and public consultations could engage fisheries, tourism, renewable energy, and coastal development more fully, and could draw on existing open forums such as the Fisheries Working Group and Habitat Working Group on Offshore Wind Energy as templates for multi-sector dialogue^83,116^.

Involving local communities in monitoring efforts integrates local observations and traditional knowledge, improving data accuracy, credibility, and fostering stewardship^117^. Adding public input mechanisms could enhance transparency and trust, allowing communities and private entities to voice concerns about national actions impacting them; for instance, by posting plain-language summaries and maintaining two-way feedback (surveys, comment trackers) through CZM’s Communications Programme pages and MORIS item listings as progress develops. While the Communications Programme currently focuses on information dissemination^115^, expanding it to facilitate two-way communication, such as online forums and public feedback updates, would connect decision-makers with the public and build stakeholder buy-in by demonstrating how feedback shapes policy within adaptive management. Moreover, to further strengthen collaboration, Massachusetts could integrate structured conflict resolution mechanisms within MSP. A standing LSI advisory council could include representatives from various municipalities/watersheds and sectors/authorities (DMF, MASSDEP, port authorities, MassCEC/offshore wind, fishing, recreation, NGOs), with outputs recorded in the OMP’s reporting cycle. This approach would formalise how competing ecological and socioeconomic interests are transparently addressed across the land-sea interface, reducing ambiguity and tying collaborative decisions to shared, up-to-date evidence.

### Component 7: Integrating principles of climate-smart planning

MSP that prioritises resilience and adaptability to climate-related impacts such as ocean warming, acidification, and sea-level rise—all of which pose significant risks to both ecosystems and economic sectors^35^—has the potential to be most impactful. Climate-smart MSP explicitly recognises climate change as a key stressor, requiring a comprehensive consideration of climate-related impacts, and strategies to minimise them in spatial planning to enhance resilience^35^. This is particularly notable in the context of multiple interacting stressors at the land-sea interface, whereby local drivers may be disproportionately impactful against this backdrop of global change. Coastal and marine ecosystems like mangroves, seagrass beds, kelp forests, and rocky shorelines act as natural buffers against storms and support biodiversity, contributing to climate adaptation^118^. Furthermore, many of these habitats play a significant role in carbon sequestration and storage, contributing to climate mitigation. Prioritising their conservation within MSP enhances adaptive capacity and promotes long-term resilience^119^.

Climate-smart MSP benefits from integrating future climate scenarios (e.g. warming, sea-level change, weather patterns), fostering cross-boundary collaboration, and using iterative, data-driven approaches to reduce climate risks and protect marine resources. Countries such as Barbados, Sweden, Mozambique, and Ireland incorporate explicit climate considerations into ocean plans, demonstrating how targeted measures like habitat restoration, flexible zoning, and data-driven modelling, support mitigation and adaptation, reduce resource-use conflicts, and strengthen socioeconomic resilience under different warming scenarios^35^.

#### How Massachusetts could operationalise Component 7

In Massachusetts, integrating climate-smart approaches into MSP is essential given the rapid regional warming in the Gulf of Maine and the state’s vulnerability to sea-level rise^72^. Programmes like the ‘StormSmart’ Coasts Programme already help municipalities adapt to these challenges by focusing on flood resilience and shoreline management guidance^91^, and planners could standardise climate assumptions through a combination of existing tools such as the Massachusetts Coast Flood Risk Model (MC-FRM) scenarios (2030/2050/2070)^120^ and the Resilient Mass plan/action trackers^121^ to drive consistent screening and updates under the Ocean Management Plan. Leveraging MassMapper/MORIS to overlay sea-level/flood layers with sensitive resources (eelgrass, shellfish, marshes) and sector footprints enables land-sea stress-testing of spatial allocations; adding marine-heatwave intensity layers and dynamic zones for migratory species (e.g. right whales) then allows scenario testing and, where appropriate, time-area adjustments (e.g. routing, timing, or speed measures) when agreed indicators exceed thresholds. Scenario planning and climate risk assessments allow anticipation of ecosystem shifts^100^, enabling planners to align blue economy activities with climate goals for greater resilience. For example, fisheries could adjust to species distribution changes due to warming waters, and coastal tourism can be managed to account for seasonal climate variability, preventing ecosystem degradation and ensuring visitor safety^122^. Furthermore, the state could incentivise climate-resilient coastal infrastructure investments, such as elevated docks and storm-resistant ports, by directing capital and grant programmes towards projects that reduce exposure and deliver adaptation co-benefits against multiple stressors, ensuring the long-term sustainability of blue economy activities at the land-sea interface.

### Component 8: Aligning existing economic and spatial planning policies

Aligning spatial planning policies across land and sea with blue economy principles is crucial for policy coherence and sustainable coastal development. This involves reviewing and harmonising existing regulations to resolve conflicts and create synergies, thereby establishing a cohesive framework that supports both environmental protection and economic growth^123^. Such coherence is essential for achieving goals like ecosystem conservation, economic development, and social equity within shared marine spaces. The European Union’s (EU) Directive on MSP (2014/89/EU)^32^ serves as a model for integrating sustainable economic development with MSP, and explicitly notes land-sea interactions. Moreover, the Directive requires member states to incorporate multidisciplinary economic, social, and environmental dimensions into their plans, aligning with the EU’s Blue Growth Strategy^124^, and the aim of the 2025 European Ocean Pact to operationalise policy alignment across environment, transport, innovation, and regional development^42^. This integration promotes balanced marine resource use, resolves sectoral conflicts, and facilitates effective cross-border cooperation.

A recent synthesis of the EU policy landscape for land-sea interactions by Innocenti and Attombri maps how marine, water, nature, and climate laws interlock and where gaps remain, particularly useful for identifying conflicts and synergies when aligning MSP with terrestrial planning and restoration agendas within a developing blue economy^125^. Streamlining regulatory pathways and simplifying permitting processes enhance compliance and adaptive governance, while mandatory stakeholder involvement ensures transparency and inclusivity. Furthermore, harmonising MSP with restoration and blue-carbon policies, such as habitat rehabilitation mandates and blue carbon initiatives, can help ensure that spatial plans not only manage uses but actively promote ecosystem recovery in degraded coastal and marine areas^126^.

#### How Massachusetts could operationalise Component 8

The Massachusetts OMP aligns local and national regulations through the CZM Federal Consistency Review, ensuring that state activities affecting the coastal zone are consistent with wider national policies^127^. There is potential to further streamline blue economy frameworks within spatial planning. Enhancing inter-agency coordination with organisations such as the Massachusetts Environmental Trust^128^ and regional programmes like the Northeast Regional Ocean Council^129^ could improve policy coherence across resource use contexts. CZM already participates in Regional Ocean Partnerships (e.g. NROC) to synchronise data and policies across New England; this forum could be used to further align multiple stressor policies at the land-sea interface more explicitly. Increasing their involvement in planning decisions would synchronise blue economy strategies with MSP, fostering a unified approach and ensuring that blue growth aligns with environmental protection. Supporting the development of a blue economy with MSP could be further encouraged through financial incentives like subsidies and certification schemes, which prioritise environmental stewardship across sectors^130^. Massachusetts could operationalise this by prioritising LSI-smart projects within existing programmes; e.g. MVP Action Grants for climate adaptation on the landward side, and MassCEC port/workforce investments tied to environmental performance for offshore-wind supply chains. Incentivising sustainable practices in user sectors such as fisheries, tourism, or renewable energy could then be linked more directly to measurable outcomes at the land-sea interface, promoting alignment between economic development and ecosystem health and resilience.

## The Future of MSP at the land-sea interface

The challenges facing coastal ecosystems are intensifying due to multiple interacting stressors, rendering traditional management approaches insufficient. The overlapping biodiversity and climate crises, driven by rising sea temperatures, ocean acidification, species loss, and feedbacks that weaken the ocean’s carbon sink capacity, underscore the urgent need for a sustainable ocean economy and holistic, adaptive management strategies across the land-sea interface^31,33^.

To achieve ecological and socioeconomic sustainability amid anthropogenic change, we propose a transformative MSP framework centred on bidirectional land-sea interactions by embedding eight complementary components into the planning processes. Notably, these components have the potential to be employed even after MSP is underway, as demonstrated by our Massachusetts case study, to yield even greater efficiency and effectiveness. Together, these elements ensure that economic activities proceed in step with ecological and resilience goals, supporting sustainable growth without sacrificing ecosystem health in the face of potentially non-linear, multiple interacting stressors. It is designed to navigate both immediate fluctuations and long‑term trends, safeguarding coastal and broader marine ecosystems, and the livelihoods they support in line with global goals such as Sustainable Development Goal 14 and the Ocean Decade Programme^131,132^.

Global discussions such as the 16th Conference of the Parties to the Convention on Biological Diversity (COP16)^133^ highlight the critical need for this approach across both land and sea. COP16 emphasised translating the Global Biodiversity Framework into actionable steps, including identifying Ecologically or Biologically Significant Marine Areas, to achieve the goal of protecting 30% of land and sea by 2030^134^. However, coastal and marine biodiversity remains underrepresented in conservation efforts, jeopardising ecosystem functions across the land-sea interface vital for carbon storage and climate regulation. Our framework bridges this gap by offering targeted, equitable coastal and marine management strategies that align with COP16 objectives. By emphasising land-sea integration, investing in data, and aligning policies, we facilitate the incorporation of marine considerations into National Biodiversity Strategies and Action Plans, aiding countries in fulfilling their biodiversity commitments.

The future success of MSP and the Blue Economy depends on adaptability to environmental uncertainties, greater focus on stressor interactions, and cross-sector innovation by bringing land and sea authorities and users to the same table. By adopting this comprehensive, adaptive, and inclusive framework, we can better navigate the complexities of multiple interacting stressors at the land‑sea interface. This integration supports international commitments like those reaffirmed at COP16 and promotes a sustainable ocean economy that meets present needs while preserving marine ecosystems for future generations.

## Supplementary information

Supplementary information

## Data Availability

No datasets were generated or analysed during the current study.
