# Supplementary material for: Balancing the blue economy and multiple stressor management in marine spatial planning at the land-sea interface
Source: NPJ Ocean Sustain. 2026 Mar 19;5(1):21. doi: 10.1038/s44183-026-00192-3 (PMC13086571; doi:10.1038/s44183-026-00192-3)
Supplement: Supplementary file 1 — Supplementary information [file 44183_2026_192_MOESM1_ESM.pdf]

International convention	Year of establishment	Summary	Multiple/cumulative stressors	Coastally specific stressors	Coastal habitat differentiation
**CBD**<br>*Convention on Biological Diversity*[1]	**Adopted:** 1992<br>**Enforced:** 1993<br><br>*Aichi targets[2]: 2010<br>*Kunming-Montreal targets[3]: 2022	*To conserve biological diversity, sustainably use its components, and ensure the fair and equitable sharing of benefits arising from the use of genetic resources.*	Kunming-Montreal Target 7: Abstractly acknowledges **the need to "consider cumulative effects"** of several pollution types. However, **lacks specification for acknowledging non-linear, interactive stressor combinations.**	Several targets acknowledge various stressors (Target 5: Wildlife harvesting; Target 7: Pollution; Target 8: Climate change).<br><br>However, they are **addressed more broadly, without direct reference to coastal zone.**	**Coastal environments somewhat differentiated** in Aichi Target 11 and Kunming-Montreal 30x30 Target 3, as well as relation to broad land-sea integration.<br><br>Generally, a greater focus on unspecified marine environments.
**UNCLOS**<br>*United Nations Convention on the Law of the Sea*[4,5]	**Adopted:** 1982<br>**Enforced:** 1994	*To establish a legal framework for all marine and maritime activities, including territorial waters, sea-bed mining, and the conservation and management of marine resources.*	**No explicit reference to the concept of multiple stressors or cumulative impacts.**<br><br>Article 192: Provides a broad mandate for the protection and preservation of the marine environment.<br><br>Article 194: Addresses reduction of marine pollution.	Article 194: Acknowledges pollution from land-based sources.<br><br>**However, addresses pollution in a broad marine sense without direct reference to coastal zone.**	Indirect reference to coastal zones more broadly by reference of pollution mitigation from land-based sources.<br><br>Generally, a greater focus on unspecified marine environments.
**UNFCCC**<br>*United Nations Framework Convention on Climate Change*[5,6]	**Adopted:** 1992<br>**Enforced:** 1994	*To stabilise greenhouse gas concentrations in the atmosphere at a level to prevent dangerous anthropogenic interference with the climate system, while promoting sustainable development and supporting adaptation and mitigation efforts.*	**No explicit reference to the concept of multiple stressors or cumulative impacts.**<br><br>Articles 3 and 4: Emphasise the need to consider interrelationships between various climate-related processes.	Article 4.1(d & e): Acknowledges that coastal areas have specific needs to be considered in climate change adaptation measures.<br><br>However, they are **addressed more broadly, without direct reference to coastal zone.**	**Coastal environments somewhat differentiated** in Article 4.1, with brief reference to integrated coastal zone management in 4.1(e).<br><br>Generally, a greater focus on unspecified marine environments.
**Paris Agreement**<br>*Subsidiary UNFCCC treaty*[6]	**Adopted:** 2015<br>**Enforced:** 2016	*To set goals to limit global warming to below 2°C, aiming for 1.5°C. The Agreement increases global adaptation, aligns financial flows with low greenhouse gas emissions and climate-resilient development, and requires submission of Nationally Determined Contributions every five years.*	**No explicit reference to the concept of multiple stressors or cumulative impacts.**<br><br>Article 7: Encourages integrated, holistic approaches to enhance adaptive capacity.	Sets broad ecosystem targets and **lacks specific reference to particularly biomes and associated context-specific stressors.**	No explicit reference to coastal zone. More general focus on **unspecified environments and ecosystems.**

Table S1: Summary of international conventions related to sustainable ocean use. Reference to multiple/cumulative stressors, coastally-specific stressors, and coastal habitat differentiation evaluated with traffic light coding system: Green = reference to topic present; Orange = partial or indirect reference; Red = absence of inadequate reference.

Key component	Foundational/operational	UNESCO MSP guide planning stage[7]
Promote research to understand stressor interactions at the land-sea interface	Foundational	NA
Integration across species, spaces, and sectoral domains	Foundational	NA
Explicitly incorporate bidirectional interactions at the land-sea interface	Foundational	NA
Invest in data collection and sharing	Operational	• Defining and Analyzing Existing Conditions (Step 5)<br>• Monitoring and Evaluating Performance (Step 9)
Prioritise adaptive, data-driven management	Operational	• Defining and Analyzing Existing Conditions (Step 5)<br>• Defining and Analyzing Future Conditions (Step 6)<br>• Monitoring and Evaluating Performance (Step 9)<br>• Adapting the Marine Spatial Management Process (Step 10)
Stakeholder engagement and cross-sectoral collaboration	Operational	• Organizing Stakeholder Participation (Step 4)<br>• Preparing and Approving the Spatial Management Plan (Step 7)<br>• Implementing and Enforcing the Spatial Management Plan (Step 8)<br>• Adapting the Marine Spatial Management Process (Step 10)
Integrate principles of climate-smart planning	Operational	• Defining and Analyzing Existing Conditions (Step 5)<br>• Defining and Analyzing Future Conditions (Step 6)<br>• Monitoring and Evaluating Performance (Step 9)<br>• Adapting the Marine Spatial Management Process (Step 10)
Align existing economic and spatial planning policies	Operational	• Preparing and Approving the Spatial Management Plan (Step 7)<br>• Implementing and Enforcing the Spatial Management Plan (Step 8)

Table S2: UNESCO MSP entry points aligned with key components.

Box 1: The Massachusetts Case Study

Massachusetts serves as an exemplary model for harmonizing Blue Economy principles with MSP. The state features a diverse marine environment with over 1,500 miles of coastline along the Atlantic Ocean and the Gulf of Maine. According to the 2024 Marine Economy Report[8], Massachusetts' marine economy comprises 5,891 businesses, supports over 86,000 employees, and generated a GDP of $8.3 billion in 2021[8]. Tourism and recreation lead the sector, employing 74% of the marine workforce and contributing 55% of marine GDP. Between 2011 and 2021, the marine economy saw a 16% GDP increase, a 41% rise in average wages, and 9% business growth, reflecting the state's commitment to economic and marine resource sustainability[8].

Massachusetts demonstrates leadership in marine conservation and sustainable growth through policies like the Massachusetts Ocean Management Plan[9,10], which integrates strategic spatial and temporal planning, and Biodiversity Conservation Executive Order No. 618[11], aligning with international biodiversity goals. Further commitment is evident in an $8 million investment in sustainable marine infrastructure and renewable energy projects, reported by the Healey-Driscoll Administration[12]. However, with the issuance of a new federal memorandum in January 2025 that places offshore wind development in limbo, the broader U.S. policy landscape remains fluid. Massachusetts has a strong state agency promoting ocean planning, as well as a robust coastal zone management agency and collaborative networks that link these with many academic institutions and research centres in the state[13–18]. Bolstered by the research, education and engagement occurring at NGOs and non-profit research and conservation organisations such as the New England Aquarium in Massachusetts (USA)[13], the state highlights the alignment of marine conservation with sustainable economic growth. Its economic significance, ecological diversity, and proactive management make it an ideal case for evaluating and operationalizing the present framework.

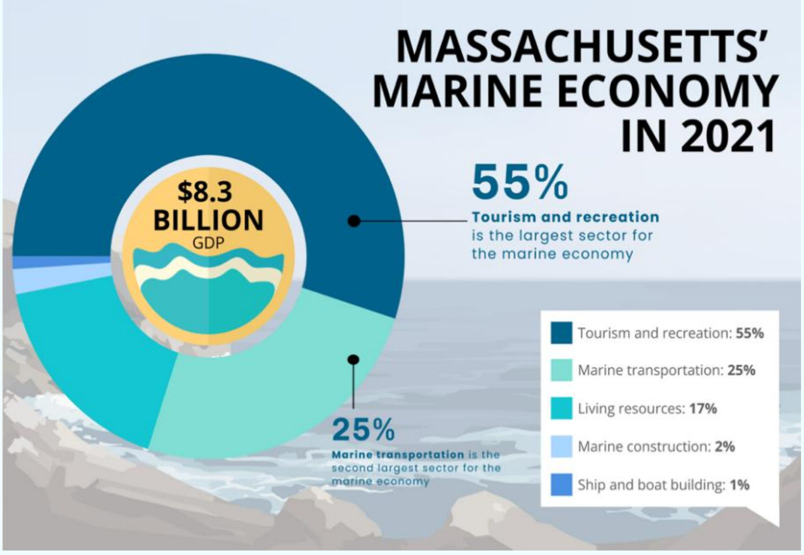

Data from NOAA 2024 Marine Economy Report: Massachusetts[8].

Figure 2 map construction and data rationale

We assembled a state-wide map of the Massachusetts land-sea interface to holistically illustrate, in a single view, (i) sensitive ecological/management features, (ii) key maritime sectors and pressures, and (iii) the Massachusetts Ocean Management Plan (OMP, 2021) regulatory context. For all data layers, we utilised live ArcGIS Feature/Map Services rather than static downloads, so the figure reflects the most current, authoritative layers and their symbology/attributes at the time of publication, while preserving more streamlined reproducibility. All Massachusetts state data used in this figure (MassGIS/CZM, DMF, MassDEP, MassDOT) are public domain under Massachusetts policy, with source acknowledgement listed below.

The map was constructed in QGIS (3.40 LTR). The project coordinate reference system used was NAD83/Massachusetts Mainland (EPSG:26986) to keep buffering and distance operations in meters. All web services (often published in WGS84 or Web Mercator) were reprojected on-the-fly. We overlaid the 2021 Massachusetts OMP Prohibited and Multi-Use management areas and used the OMP Planning Area Mask as the map frame, to align subsequent sectoral/biological layers with current plan zoning and see where uses are explicitly constrained or allowed. To further populate the scope of the OMP, we illustrate a selection of key regulatory biomes within the remit of the OMP, including:

- Intertidal flats.
- Concentrated commerce traffic.
- Concentrated recreational boating.
- Important fish resources.

We chose a selection of three sensitive ecological/management features of state-wide policy relevance to visualise, with complementary socio-ecological relevance across the nearshore including:

- MassDEP Eelgrass, Phase 6 (2019-2023) polygons (most recent eelgrass mapping campaign illustrating wider seagrass habitat condition).
- Designated Shellfish Growing Areas (DMF) polygons (harvest classifications maintained by the Division of Marine Fisheries, demonstrating water quality/food safety management domains). We collapsed the five DMF classes (Approved, Conditionally Approved, Restricted, Conditionally Restricted, Prohibited) into three legend bins; Open (Approved + Conditionally Approved), Restricted (Restricted + Conditionally Restricted), and Prohibited, to present status intelligibly at state-wide scale while preserving regulatory meaning.

- North Atlantic Right Whale Seasonal Management Areas (SMAs) polygons (federal mandatory seasonal speed-restriction zones to reduce ship strikes, illustrating protected-species risk management).

We similarly mapped representative layers to represent key sectors and pressures at the land-sea interface that occupy meaningful sea space, are commonly referenced in MSP and coastal permitting, and have robust, up-to-date state-wide coverage:

- NOAA ENC "Shipping Lanes & Regulations" for traffic corridors (TSS, fairways, recommended routes) and special routeing areas (Areas To Be Avoided, Particularly Sensitive Sea Areas, Precautionary Areas). NOAA's service is extracted from official ENCs and is updated weekly, making it the most current public view of federal routeing measures. We clipped the national layer to the OMP mask for performance. For readability, we grouped sub-types into (i) traffic corridors (Traffic Separation Schemes/Traffic Lanes, Shipping Fairways Lanes & Zones, Recommended Routes) and (ii) special routeing areas (ATBA, PSSA, Precautionary), because individual subtype symbology is visually dense at state-wide scale, while providing limited additional insight for our purposes.
- BOEM Offshore Wind Leases (OCs lease outlines) to situate present federal renewable-energy footprints.
- USACE historical dredge footprints (polygons to 1998) to depict legacy benthic disturbance in channels/anchorages/harbours; included as a spatial proxy of past alteration of seabeds and inlets.
- MassDEP Combined Sewer Overflow (CSO) outfalls to highlight land-derived pollution nodes near the coastal margin.

Moreover, to further demonstrate the scale of activity at the coastal interface and the incidence of tourism/recreation, we added datasets maintained by state agencies to provide intuitive proxies for where people interact with the coast, including:

- Marine Beaches (public and semi-public)
- Marinas (most recent update, 2019)
- Seaports

Licencing and permissions:

MassGIS indicates that its datasets are in the public domain and may be used for any purpose with appropriate acknowledgement. U.S. federal datasets used (NOAA, BOEM, USACE) are produced by U.S. Government agencies and are made available for reuse; NOAA Office of Coast Survey data are publicly released under a CC0 public-domain dedication. Figure 2 contains no third-party copyrighted base-map imagery or proprietary cartographic products; the map layout, symbology, and any aggregation were created by the authors.

Data sources/web feature services used are as follows:

- Eelgrass (MassDEP Phase 6, 2019-2023): https://www.arcgis.com/home/item.html?id=06aaf0fdd9f54a11b815a169fde88989
- Designated Shellfish Growing Areas (DMF): https://arcgisserver.digital.mass.gov/arcgisserver/rest/services/AGOL/DSGA/FeatureServer
- Right Whale Seasonal Management Areas (NOAA): https://services2.arcgis.com/C8EMgrsFcRFL6LrL/arcgis/rest/services/Seasonal_Management_Areas/FeatureServer/3
- ENC “Shipping Lanes & Regulations” (NOAA): https://encdirect.noaa.gov/arcgis/rest/services/NavigationChartData/MarineTransportation/FeatureServer/0
- BOEM Offshore Wind Leases: https://services7.arcgis.com/G5Ma95RzqJRPKsWL/ArcGIS/rest/services/Wind_Lease_Boundaries__BOEM_/FeatureServer
- USACE Dredge Projects (historical): https://services1.arcgis.com/7iJyYTjCtKsZS1LR/arcgis/rest/services/Dredge_Projects_USACE_HIstorical_to_1998/FeatureServer
- MassDEP CSOs: https://services1.arcgis.com/7iJyYTjCtKsZS1LR/arcgis/rest/services/MassDEP_CSOs_2/FeatureServer/0
- Marine Beaches: https://arcgisserver.digital.mass.gov/arcgisserver/rest/services/AGOL/Marine_Beaches/FeatureServer
- Marinas (2019): https://services1.arcgis.com/7iJyYTjCtKsZS1LR/arcgis/rest/services/Marinas_2019/FeatureServer

- Seaports: https://gisstg.massdot.state.ma.us/arcgis/rest/services/Multimodal/Seaport/MapServer/0
- Massachusetts OMP (2021): https://services1.arcgis.com/7iJyYTjCtKsZS1LR/arcgis/rest/services/OMP_2021_18Nov2021/FeatureServer
- New England state outlines: https://arcgisserver.digital.mass.gov/arcgisserver/rest/services/AGOL/NewEnglandStates/FeatureServer

Supplementary references

1. Convention on Biological Diversity. in *United Nations Biodiversity Conference: COP 16, Cartagena Protocol COP/MOP 11, Nagoya Protocol COP/MOP 5.* (Secretariat of the CBD, 2024).
2. Conference of the Parties to the Convention on Biological Diversity (CBD). *Aichi Biodiversity Targets*. (2010).
3. Conference of the Parties to the Convention on Biological Diversity. *Kunming-Montreal Global Biodiversity Framework*. (2022).
4. United Nations. *United Nations Convention on the Law of the Sea*. (1982).
5. United Nations. *United Nations Framework Convention on Climate Change*. (1992).
6. United Nations. *Paris Agreement* . (2015).
7. Ehler, Charles, Douvere, Fanny & Ioc. *Marine Spatial Planning: A Step-by-Step Approach toward Ecosystem-Based Management; IOC. Manuals and Guides; Vol.:53; 2013*. (2009).
8. NOAA Office for Coastal Management. 2024 Marine Economy Report - Massachusetts. (2024).
9. Massachusetts Executive Office of Energy and Environmental Affairs. *2021 Massachusetts Ocean Management Plan: Volume 1 - Management and Administration*. (2021).
10. Massachusetts Executive Office of Energy and Environmental Affairs. *2021 Massachusetts Ocean Management Plan: Volume 2 - Baseline Assessment and Science Framework*. (2021).
11. Maura Healey. *Executive Order No. 618*. (Commonwealth of Massachusetts, Executive Department, Boston, 2023).
12. Massachusetts Executive Office of Economic Development & Seaport Economic Council. Healey-Driscoll Administration Announces $8 Million in Grants to Support Massachusetts' Blue Economy. https://www.mass.gov/news/healey-driscoll-administration-announces-8-million-in-grants-to-support-massachusetts-blue-economy (2023).
13. Anderson Cabot Center for Ocean Life: New England Aquarium. https://www.neaq.org/conservation-and-research/anderson-cabot-center-for-ocean-life/.
14. Woods Hole Oceanographic Institution. https://www.whoi.edu/.
15. Marine Biological Laboratory: University of Chicago. https://www.mbl.edu/.
16. Urban Harbors Institute: UMass Boston. https://www.umb.edu/uhi/.
17. Massachusetts Office of Coastal Zone Management (CZM). https://www.mass.gov/orgs/massachusetts-office-of-coastal-zone-management-czm.
18. Northeast Regional Ocean Council. https://www.northeastoceancouncil.org/.

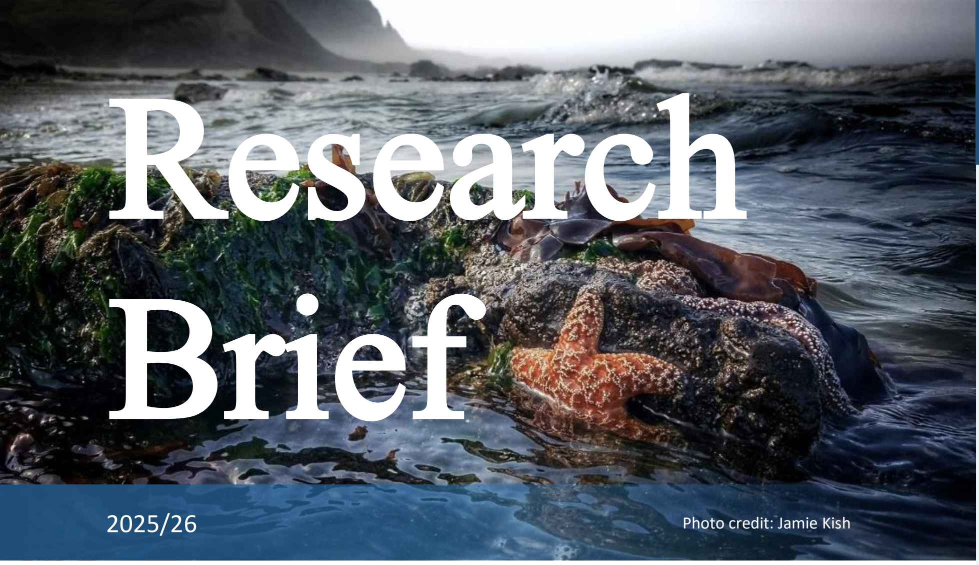

Photo credit: Jamie Kish

Balancing the Blue Economy and Multiple Stressor Management in Marine Spatial Planning at the Land-Sea Interface

Massachusetts could serve as a case-study for balancing Marine Spatial Planning and Blue Economy initiatives to support coastal resilience and sustainable growth.

Coastal ecosystems provide many services, including biodiversity, coastal protection, and supporting local economies. However, these ecosystems face increasing local-level stressors, compounding the effects of global stressors in unpredictable ways across the land-sea interface. What happens at the coastal zone affects all interconnected life across the land-sea interface. With the combined impacts of co-occurring human stressors mounting, we must develop actionable strategies at different spatial and temporal scales to mitigate these impacts and protect these critical ecosystems.

In the United States, the National Strategy for a Sustainable Ocean Economy (NSSOE) and the Ocean Climate Action Plan (OCAP) provide comprehensive frameworks for marine coastal management. As one of 18 nations in the High-Level Panel for a Sustainable Ocean Economy, the US has demonstrated its commitment to sustainable ocean management practices within blue economy frameworks. Aligning Marine Spatial Planning (MSP) with Blue Economy initiatives can synergize economic and environmental outcomes across at the land-sea interface. This is particularly beneficial for coastal shorelines, representing an intricate network of sectoral activities across land and sea, such as fishing, tourism, and offshore renewable energy development. By managing the spatial distribution of uses, MSP helps ensure that resource use contributing to the Blue Economy is sustainable and equitable, supporting the resilience and prosperity of coastal ecosystems and the communities that rely upon them.

Massachusetts offers a valuable case-study for harmonizing MSP goals with blue economy initiatives. Its 1500+ mile coastline is a dynamic intersection of biodiversity and associated uses, including commercial fishing, port activities, and tourism.

KEY POINTS FOR POLICYMAKERS

Invest in data collection and sharing

Increase investment in standardized data collection and sharing methods that track multiple stressors at coastal zones and explicitly link land-to-sea with sea-to-land indicators. Coordinated data efforts across state and federal agencies could facilitate evidence-based decision-making and ensure that policies are informed by the most accurate site-specific ecological and socio-economic data. Efforts should also support research into knowledge gaps on emerging stressors and encourage the development of predictive tools and interaction-aware research and analysis.

Prioritize iterative monitoring and management

Implement flexible and dynamic governance strategies that allow adaptive responses to coastal ecosystems' unique and changing nature, and their uses. Marine Spatial Planning frameworks should incorporate iterative monitoring and management, enabling policies to adjust in response to new data and research insights on the cumulative and potentially disproportionate effects of stressors, such as coastal erosion, nutrient runoff, and the broader impacts of climate change.

Integrate across species, spatial, and sectoral management domains

Develop and align policies to explicitly address the integration of land-based and marine impacts, particularly at the coastal zones where human activities overlap intensely. This could be facilitated through a 'species-spaces-sectors' (3S) approach, whereby interactions between these domains are holistically addressed across the broader ecosystem. This could help ensure that spatial planning measures are environmentally and economically effective across the land-sea interface, supported further by shared mapping services and decision-making that convene multisector stakeholders.

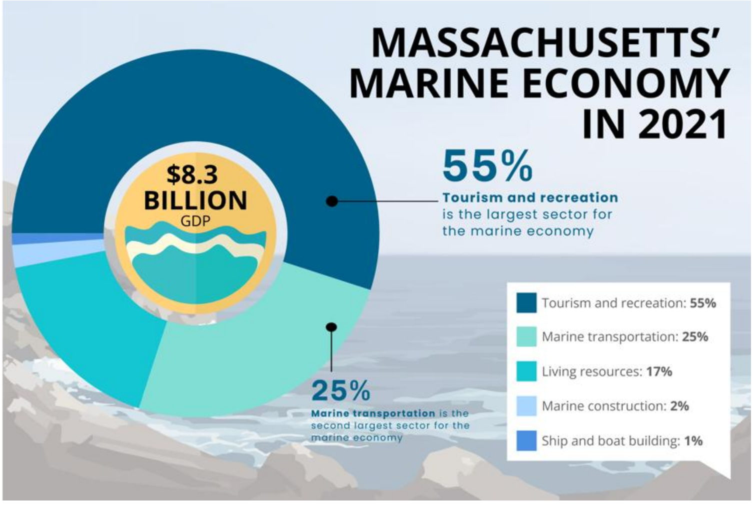

State-level policies and programs, including the Ocean Management Plan (OMP) and Coastal Habitat Programme (CHP) address ecological conservation and sustainable economic growth. While the state's existing frameworks offer significant protection for broader coastal areas, the explicit focus on shoreline differentiation and their unique challenges could be enhanced. Strengthening these policies to address the idiosyncrasies of the coastal zone more directly would further ensure that these critical habitats receive targeted attention and effective management strategies. Furthermore, expanding current single-stressor-oriented initiatives, such as the Coastal Pollutant Remediation Grant Program, to emphasize interactions of local human impacts against a backdrop of global change, could enhance the resilience and productivity across the land-sea interface.

Massachusetts spatial planning frameworks are well positioned to effectively integrate spatial tools with Blue Economy initiatives. The OMP can act as a principal planning framework, which already delineates specific zones for various use activities, helping to ensure economic use projects are strategically located to minimize environmental impacts. There is an opportunity to align these spatial zones further with Blue Economy initiatives to create a more cohesive management strategy rooted in holistic sustainability. Programs and initiatives can effectively utilize spatial planning tools like GIS (Geographic Information Systems) for habitat zoning to designate economically sustainable multi-use areas. Combined with the Massachusetts Coastal Zone Authority's emphasis on adaptive, data-driven management, incorporating these tools creates a solid foundation for strategically managing resources across species, spaces, and sectors. A spatially-optimised approach can enhance the placement of economic activities while simultaneously flagging interactive stressors and encouraging coastal resilience.

The expected outcomes of Marine Spatial Planning, Blue Economy objectives, and multiple stressor management are significant. This integrated approach could greatly facilitate the management of multiple stressors, allowing sensitive coastal ecosystems to withstand environmental changes and human impacts better. Sustainable economic growth may be fostered by ensuring that ocean activities are aligned with ecosystem health, thereby securing the long-term viability of sectors. Massachusetts is positioned to use its existing management plans in tandem with Blue Economy initiatives underway in the Commonwealth to effectively manage and protect coastal ecosystems. By addressing the unique needs of its coastal zone and the communities that rely upon them, the state clearly demonstrates potential for fostering a truly sustainable Blue Economy against a backdrop of both local and global change.

ABOUT THE AUTHORS

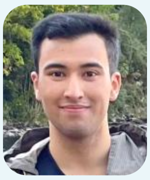

Ramesh Wilson is a PhD (DPhil) Candidate at the University of Oxford, and lead author of this study and accompanying Research Brief. He researchers the cumulative, non-linear impacts of multiple stressors on global rocky shore ecosystems, focussing on scalable, cost-effective experimental research, with practical management implications at the land-sea interface.

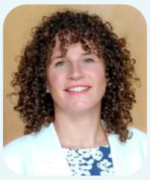

Sarah Reiter, MS, JD is Associate VP of Ocean Conservation Practise at the New England Aquarium. She leads initiatives related to biodiversity, climate resilience, and sustainable and equitable practises. She also holds a position as Adjunct Professor of Law at Vermont Law and Graduate School.

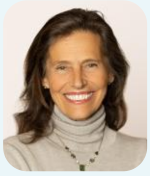

Dr Tundi Agardy is the founder and director of Sound Seas, an organization working at the nexus of marine science and policy. She was formerly Distinguished Visiting Fellow at Worcester College, University of Oxford. Tundi's research primarily focuses on the sustainability of coastal ecosystems, restoration science, biodiversity conservation, marine spatial planning, and institutional frameworks for integrated ecosystem-based management.

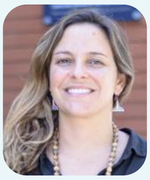

Dr Catarina Frazão Santos is an Associate Professor at CIÊNCIAS, University of Lisbon, Awardee of the European Research Council (ERC Starting Grant), and Honorary Research Associate at the University of Oxford. Dr Frazão Santos research focuses on the challenges of developing sustainable ocean planning and governance under global environmental and social change.

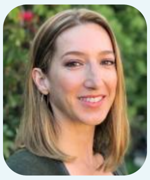

Dr Lisa Wedding is Principal Investigator of the Oxford Seascape Ecology Lab, Associate Professor in Physical Geography, and a Tutorial Fellow of Worcester College, Oxford University. Dr Wedding leads research focussing on understanding the socio-ecological causes and consequences of spatial patterns and ecological processes in the marine environment.

This briefing accompanies the following article:

Balancing the Blue Economy and Multiple Stressor Management in Marine Spatial Planning at the Land-Sea Interface.

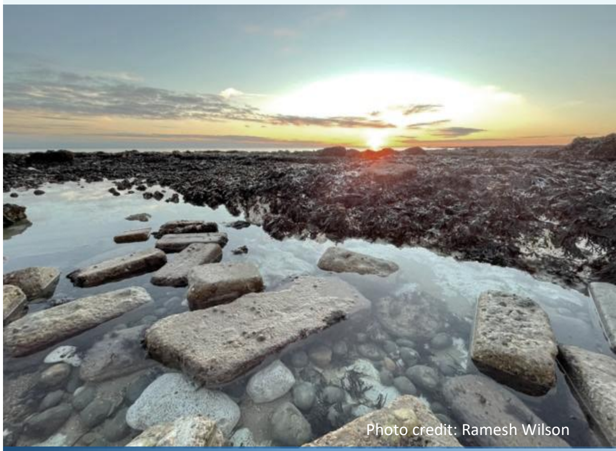
Photo credit: Ramesh Wilson

FOR MORE INFORMATION

Contact Ramesh Wilson:
rameshwilson14@gmail.com
